# Development of a Low-Cost and Easy-Assembly Capillary Electrophoresis System for Separation of DNA

**DOI:** 10.3390/bioengineering12030303

**Published:** 2025-03-17

**Authors:** Jiawen Li, Shuaiqiang Fan, Jiandong Zhu, Bo Yang, Zhenqing Li, Dawei Zhang, Yoshinori Yamaguchi

**Affiliations:** 1 Tianjin Key Laboratory of Retinal Functions and Diseases, Tianjin Branch of National Clinical Research Center for Ocular Disease, Eye Institute and School of Optometry, Tianjin Medical University Eye Hospital, 251 Fu Kang Road, Tianjin 300384, China; 2 Shanghai Key Lab of Modern Optical System, Engineering Research Center of Optical Instrument and System, Ministry of Education, University of Shanghai for Science and Technology, No. 516 JunGong Road, Shanghai 200093, Chinayangbo419@usst.edu.cn (B.Y.); dwzhang@usst.edu.cn (D.Z.); 3 Picotecbio-Waseda Joint Research Lab, Comprehensive Research Organization, Waseda University, NishiTomita, Honjo 367-0035, Japan; yoshi.yamaguchi@ap.eng.osaka-u.ac.jp; 4 Department of Applied Physics, Graduate School of Engineering, Osaka University, Osaka 565-0871, Japan

**Keywords:** capillary electrophoresis, laser-induced fluorescence, polymerase chain reaction, nucleic acids, SYBR Green I, SYBR Green I, Gel Green, EvaGreen, hydroxyethyl cellulose

## Abstract

Capillary electrophoresis based on laser-induced fluorescence (CE-LIF) plays an important role in the analysis of nucleic acids. However, the commercial CE-LIF is not only quite expensive but also inflexible, thus hindering its widespread use in the lab. Herein, we proposed a compact, low-cost, and flexible CE-LIF system. We also investigated its stability by separating the DNA ladders. Experiments demonstrated that the relative standard error of the relative fluorescence intensity and migration time was lower than 6.2% and 1.1%, respectively. The aperture size of the light source illuminating the capillary can affect the separation performance. Smaller apertures offer higher resolution length for the adjacent DNA fragments but may reduce the number of theoretical plates. Various fluorescent dyes (e.g., SYBR Green I, Gel Green, EvaGreen) can be employed in the self-built system. The limit of detection of dsDNA was as low as 0.05 ng/μL. The working range for DNA was 0.05 ng/μL~10 ng/μL. Finally, we have successfully separated the PCR products of the target gene of *Porphyromonas gingivalis* and *Candida albicans* in the home-built CE system. Such a robust CE-LIF system is easy to assemble in the lab. The total cost of the assembled CE system did not exceed 1100 USD. We believe this work can advance the application of CE and hope it will facilitate the easy assembly of flexible CE instruments in labs.

## 1. Introduction

Capillary electrophoresis (CE) is a liquid separation technique that utilizes a capillary as the separation channel. Under the influence of an electric field, particles migrate toward either the anode or cathode based on their charge. The separation is achieved due to differences in the particles’ charge, size, and interaction with the buffer solution. It has the advantages of fast speed, low sample consumption, and high automation. Therefore, it has been widely applied in the field of bioanalytical chemistry [1,2,3,4].

To date, there have been many technologies combined with CE for the detection of samples, including electrochemical detection [5,6], UV absorbance detection [7,8], inductively coupled plasma mass spectrometry (ICP-MS) [9,10], and laser-induced fluorescence (LIF) [11,12,13]. Among these methods, UV absorption and LIF are the two main methods employed for the analysis of nucleic acids or protein. Compared with the former one, CE-LIF has the advantages of higher sensitivity and fast response, and it was widely employed in the field of biochemistry. For example, Morani et al. elucidated the extracellular vesicles’ electrokinetic distribution [14,15]. Ban’s group has performed an accurate miRNA quantitation in crude cell lysate by CE-LIF. Song’s lab has used it to study the pharmacokinetic distribution of miRNA-497 as a model miRNA for a lung target [16]. However, all of the work above was finished by a commercial PA800 plus CE system. Although it is quite convenient, it lacks flexibility because the wavelength of the light source and the length of the capillary are fixed. Moreover, the high price hindered its widespread application, especially for labs in the developing countries.

To enhance flexibility, reduce costs, and improve sensitivity, researchers have dedicated significant efforts to the development of compact CE systems. For example, Breadmore et al. developed a miniaturized capillary electrophoresis (CE) system integrated with an LED absorption detector for metal ion detection, and they realized detection of Co(II), Cu(II), Cu(II), and Zn(II) after complexation with 4-(2-Pyridylazo) resorcinol [17]. Hauser’s lab assembled a CE instrument based on commercial components and realized separating different metal ions [18,19]. Pan JZ’s group used a minimal system and low-cost instrument components. Although the miniaturized high-speed CE analyzer size reduced to 90 × 75 × 77 mm, it sacrificed the limit of detection [20].

In the past few years, we have carried out a series of studies to improve the separation performance of dsDNA, RNA and proteins. For example, we proposed the square-wave pulsed field CE (SWCE) and found that DNA fragments smaller than 1000 bp can be resolved at lower modulation depth (<50%), while the high modulation depth (>100%) favors the separation of larger DNA fragments (>1000 bp) [21]. However, the large and small DNA fragments cannot be simultaneously revolved with high efficiency. To overcome this problem, we proposed the inversion pulsed field CE (IFCE) [22], which can resolve the DNA fragments smaller than 10,000 bp with high resolution. The main difference between SWCE and IFCE is the pulsed field and pulsed duration of the electric field. Moreover, we also studied the factors that may improve the separation efficiency of RNA, proteins, and PCR products.

Based on the above work, we herein developed a flexible and low-cost CE-LIF system. We also investigated the factors that may influence the separation performance by separating the DNA ladders. *Porphyromonas gingivalis* (*P.g*) is a Gram-negative rod-shaped bacterium, which is an important pathogen in the oral cavity and is strongly associated with periodontal disease. *Candida albicans* is a kind of fungus that belongs to the genus Candida. It is one of the most common pathogenic fungi in clinical practice. To further validate its practicality, we have detected these two micro-organisms in the home-built system. Such a system is easy to assemble in the lab and versatile with respect to the analysis of nucleic acids and micro-organisms. It may be of great value for researchers in developing countries and could help them to build the home-built CE system.

## 2. Materials and Methods

### 2.1. Chemicals

The 50 bp DNA ladder is from Solarbio (Beijing, China). The DNA ladder consists of 8 DNA fragments between 50 and 500 bp in multiples of 50 bp. The 250 bp DNA fragment is brighter than the other DNA fragments and serves as a visible reference indicator. The 5× TBE buffer (Tris-Borate-EDT), ultrapure water, and 10000× SYBR Green I were bought from Solarbio (Beijing, China). The TBE buffer was diluted with ultrapure water at a ratio of 1:9 to prepare a 0.5× TBE buffer solution, which was then stored for future use. The 20× EvaGreen and 10,000× Gel Green were purchased from Biotium (Fremont, CA, USA). Both SYBR Green I and Gel Green were separately diluted in ultrapure water to 100× as a store solution. The stock solution of EvaGreen was prepared according to the same protocol. Hydroxyethyl cellulose (HEC) was purchased from Sigma-Aldrich (Shanghai, China). SpeedSTAR HS DNA Polymerase was bought from Takara (Beijing, China). The background electrolyte for CE consisted of 0.5× TBE, 0.5% HEC, and fluorescent dye.

The electrolyte buffer was prepared by dissolving HEC powder into the 0.5× TBE solution. In order to dissolve it completely, the solution was placed on a magnetic stirrer for one week. Before CE, the reserve Gel Green was added into the prepared HEC solution, and the final concentration of Gel Green was smaller than 20×.

### 2.2. Amplification of Target Genes by PCR

PCR was carried out in a T-100 thermal cycler (Bio-Rad, Hercules, CA, USA). The forward and backward primers for *Porphyromonas gingivalis* were TGTAGATGACTGATGGTGAAAACC and ACGTCATCCCCACCTTCCTC, respectively. While the primers corresponding to *Candida albicans* were TCCGTAGGTGAACCTGCGG and TCCTCCGCTTATTGATATGC, respectively. The size of the amplicons was 197 bp and 500 bp. The primers were synthesized by Sangon Biotech (Shanghai, China). The PCR mixture consisted of 1.0 μL sample and 49 μL reaction buffer, which was composed of 5.0 μL 4.0 μL dNTP mixture (2.5 mM), 10× Fast Buffer I, 0.25 μL SpeedSTAR HS DNA Polymerase, and 200 nM primers. The amplification conditions consisted of 95 °C for 10 s followed by 40 cycles of 95 °C for 10 s (denaturation) and 64 °C for 30 s (annealing and extension). The negative control was performed by heating the PCR solution without a template. Prior to CE, the PCR products were stored in a refrigerator for use.

### 2.3. Construction of Small Capillary Electrophoresis System

**Figure 1:**
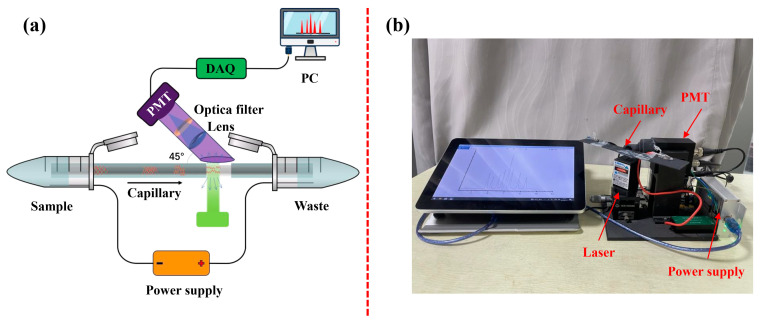
The (**a**) schematic and the (**b**) prototype of the self-built capillary electrophoresis system. DAQ: data acquisition; PMT: photomultiplier tube.

The compact CE system (Figure 1) built in our lab consisted of a 505 nm laser diode (LD, Oxlaser, Shanghai, China) as a light source, a mini high-voltage power supply (KDHM-G-24S8000N-V, Corso Electronic, Xi’an, China), an ID/OD = 75/365 (μm/μm) fused-silica capillary (Polymicro Technologies, Phoenix, AZ, USA), a H7827-011 photomultiplier tube (PMT, Hamamatsu Photonics, Tokyo, Japan), and a DAQ122 Data Acquisition Card (Lockzhiner Electronic, Fuzhou, China). Theoretically, the light from the LD was filtered by an optical filter (see Figure S1 in the Supporting Information) and then entered into the detection window of the capillary. The fluorescence light from the DNA-SYBR Green I conjugate was filtered by another optical filter (see Figure S2 in the Supporting Information). To reduce the background noise, the fluorescence was collected by a lens at an angle of 45° to the emission direction of LD, and subsequently, it entered into the PMT. Finally, the optical signal was processed by DAQ122. The wavelength of the light source and the optical filter were selected based on the fluorescent dye. The above components were assembled in a dark box. Each experiment was performed five times for repeatability.

## 3. Results and Discussion

### 3.1. Stability of the System

**Figure 2:**
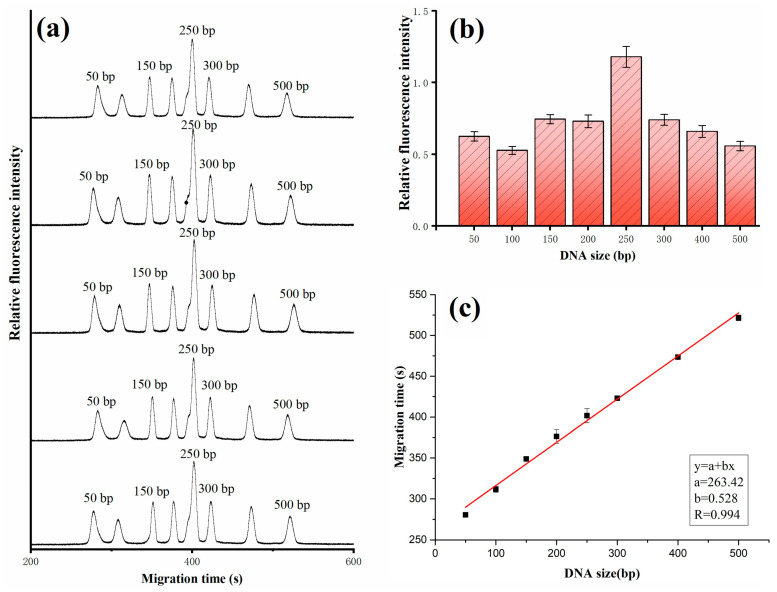
(**a**) The electropherogram of 50 bp DNA ladder based on the self-built CE system, (**b**) the relative fluorescence intensity, and (**c**) the migration time for each DNA fragment. Electrophoretic conditions: 0.5% HEC, 1300 k; injections, 100 V/cm, 2 s; separations, 100 V/cm. The total length and effective length of the capillary were 10 cm and 8 cm, respectively.

To verify its stability, multiple separations of 50 bp DNA ladder (90 ng/μL) were performed under the same electrophoretic conditions. The electric field strength was 100 V/cm. The sieving polymer consists of 0.5% HEC (1300k) and 2× SYBR Green I. The background electrolyte was 0.5× TBE. The effective length and the total length of the capillary were 8 cm and 15 cm, respectively. The samples were electrokinetically introduced into the capillary at 100 V/cm for 2.0 s. To avoid cross-contamination, the capillary was rinsed with ultrapure water with a vacuum pump for 3 min prior to and after each run; then, the sieving medium was pumped into the capillary. The repeatability was assessed by comparing the migration time and fluorescence intensity corresponding to the peak. The fluorescence intensity was obtained by calculating the area integration of the peak. The results showed that eight DNA fragments, ranging from 50 bp to 500 bp, were baseline resolved within 10 min (Figure 2a). The relative standard deviation (RSD) of the fluorescence intensity for each peak was between 5% and 6.2% (Figure 2b), and the RSD of the migration time was less than 1.1% (Figure 2c). Detailed information about the RSD is listed in Table 1. There is a linear relationship between the DNA size and the migration time, and the correlation coefficient R between them was 0.98, indicating that the system can be used for the determination of DNA size.

### 3.2. The Effect of the Aperture of the Light Source

Resolution (*R*) and theoretical plate number (N) are two important factors widely employed in chromatography and other separation techniques, which can be applied to evaluate the separation performance. *R* is defined as $R=\frac{2(t_{R_{2}}-t_{R_{1}})}{{(W}_{1}+W_{2)}}$, where *t* and *W* represent the migtation time and half width of the peak in the electroperogram. A higher resolution indicates a better separation. For example, *R* > 1 means the two peaks are almost completely resolved. However, it is difficult to evaluate the separation performance if the size difference between the adjacent DNA fragments varies greatly. Generally, R for 100 bp and 200 bp was smaller than *R* for 1000 bp and 10,000 bp under the same electrophoretic conditions, but we cannot conclude that the separation performance for 100 and 200 bp was lower than that of 1000 bp and 10,000 bp for a certain CE system. Thus, we applied resolution length (RSL) to evaluate the separation performance, which is determined by RSL = Δn/R. This can give the minimum DNA size separated by CE, where Δn is the size difference between the adjacent peaks in the electropherogram. A higher value of N indicates that the peak in the electropherogram was narrower, which tends to obtain a higher value of *R*.

**Figure 3:**
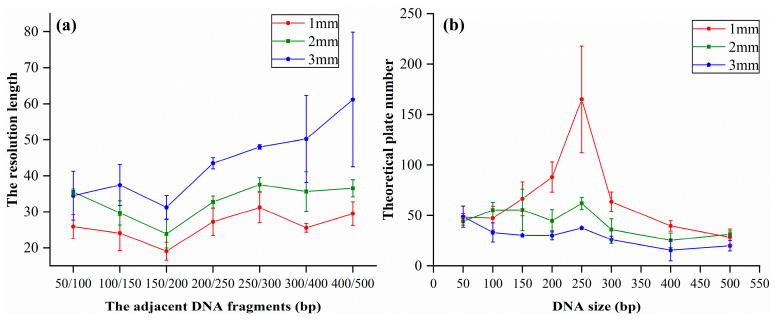
The (**a**) resolution length and (**b**) theoretical plate number of 50 bp DNA ladder.

Although the light emitted from the LD with the larger size may enhance the fluorecence intensity, it can also increase the background noise because the diameter of the capillary is quite small. Consequently, the resolution of the adjacent DNA fragments was reduced. Therefore, we investigated the effect of the aperture of the light on the resolution by separating the DNA ladders. Prior to CE, the DNA ladders were diluted by 3%. The electrophoretic conditions were the same as the ones in Figure 2, and the diameter of the aperture was 1 mm, 2 mm, and 3 mm, respectively. Data in Figure 3a revealed that RSL decreased with the increase in the aperture or DNA size, indicating that the resolving ability had deteriorated. Moreover, we found that the number of theoretical plates was larger if the diameter of the aperture was quite small (Figure 3b). Thus, we can conclude that we need to make the aperture of the light smaller if we want to improve the resolution, although this may decrease the fluorescence intensity. Therefore, we used a 1 mm aperture in the home-built CE system.

### 3.3. The Effect of the Fluorescent Dyes and Limit of Detection

**Figure 4:**
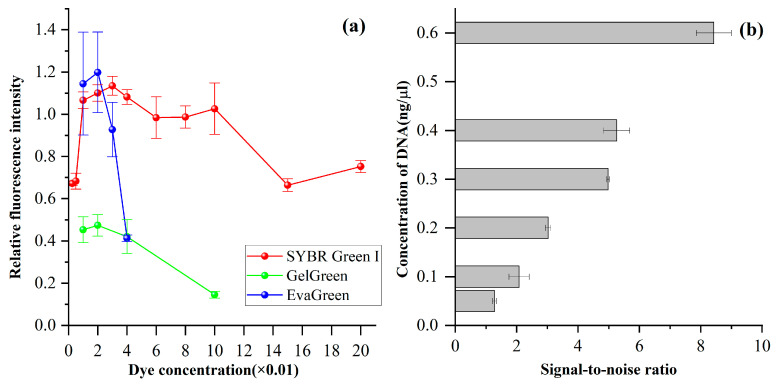
(**a**) The relative fluorescence intensity corresponding to different dyes and (**b**) the limit of detection.

SYBR Green, Gel Green, and Eva Green are three popular fluorescent dyes employed in electrophoresis because the conjugate of these dyes and DNA can be excited by visible light, which poses no harm to human health. They are unlike ethidium bromide, which is excited by ultraviolet light. We investigated the fluorescence intensity by separating 50 bp DNA ladder with varied concentrations of these fluorescent dyes. The electrophoretic conditions were as follows: 0.5% HEC (1300k) in 0.5× TBE; electric field strength for sample injection and separation (100 V/cm). The sieving polymer consists of 0.5% HEC (1300k) and 2× SYBR Green I. The background electrolyte was 0.5× TBE. The effective length and the total length of the capillary were 8 cm and 15 cm, respectively. The excitation and emission spectra corresponding to these three dyes with the DNA complex are 494/521 nm, 500/530 nm, and 499/526 nm, respectively. The effect of the fluorescent dyes was evaluated by measuring the relative fluorescence intensity (RFI) corresponding to the 250 bp DNA. Results demonstrated that RFI first increased with the concentration of fluorescent dyes; then, it decreased with the increase in concentration (Figure 4a). This is possibly because at higher concentrations, dyes might start to aggregate or form complexes, and the complex of DNA and dye cannot efficiently emit light in the same way as individual dye molecules. The optimal concentration of SYBR Green I, Gel Green, and Eva Green was 3×, 2×, and 2×, respectively. Furthermore, we also noticed that the migration times of DNA increased with the increase in the concentration of dyes, and this is possibly because the mass-to-charge of the DNA–dye conjugate changed. The molecular weight for SYBR Green I, Gel Green, and EvaGreen was 509.7 g/mol, 1198.43 g/mol, and 1119.06 g/mol, respectively.

Limit of detection (LOD) determines the minimum volume of nucleic acid that can be detected. Thus, we determined LOD by resolving the 50 bp DNA ladder and assessed it by calculating the signal-to-noise ratio (SNR). The DNA sample was injected into the capillary at 100 V/cm for 2 s. The SNR was calculated based on the fluorescence intensity of 100 bp DNA fragment and the baseline. The LOD was 0.2 ng/μL (SNR = 3). It showed that SNR increased with the increase in DNA concentration, and SNA was larger than 1, even if the DNA concentration was 0.05 ng/μL. Experiments demonstrated that the DNA ranged from 0.05 ng/μL to 10 ng/μL, which can be well resolved by the home-built CE system.

### 3.4. Application of the Self-Built CE System for Identification of Micro-Organisms

**Figure 5:**
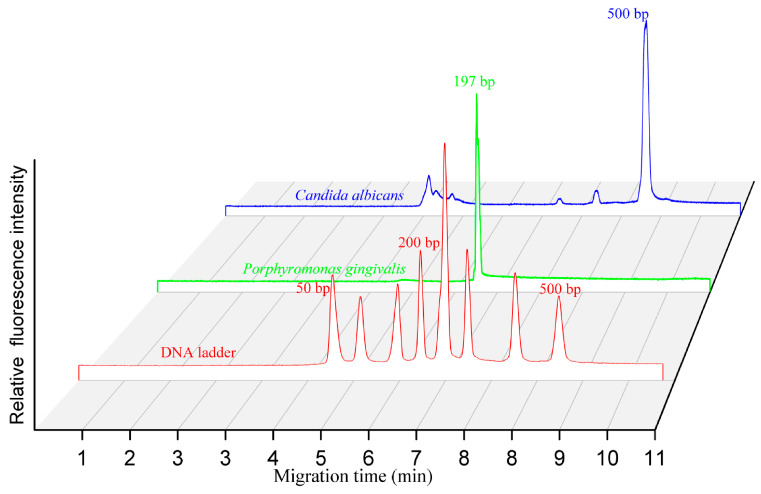
Separation of PCR products in the self-built capillary electrophoresis system. Electrophoretic conditions: 0.5% HEC, 1300 k; injections, 100 V/cm, 2 s; separations, 100 V/cm. The total length and effective length of the capillary were 10 cm and 8 cm, respectively.

To validate its practicality, we have resolved the PCR products of *P.g* and *Candida albicans*. The former one is one of the main periodontal pathogens that contribute to chronic periodontitis, while the latter one widely exists on the mucous membranes of the human. For example, *Candida albicans* is responsible for vaginal yeast infections when it is out of balance with the healthy bacteria in the body. It is amplified in a T-100 thermal cycler. The size of the amplicon was 197 bp and 500 bp, respectively. We have separated the PCR products in 0.5% HEC (1300k). The electric field strength for separation and sample injection was 100 V/cm. The results show that DNA fragments smaller than 500 bp were well resolved within 550 s (Figure 5), and the migration times corresponding to the PCR products of *Candida albicans* (500 bp) and *P.g* (197 bp) matched well with the DNA ladders, indicating that such a system is reliable for the identification of micro-organisms. Moreover, no peak was observed corresponding to the negative control.

## 4. Conclusions

This work developed and validated a compact, low-cost, and flexible home-built CE-LIF system for nucleic acid detection. The system mainly consisted of a portable power supply, a laser diode as the light source, two optical filters, and a PMT for fluorescence detection. We evaluated the stability of the system by resolving 50 bp DNA ladder in HEC as a sieving buffer. We also investigated the RSD of fluorescence intensity and migration time and obtained the optimal size of the aperture and the concentration of SYBR Green I, Gel Green, and EvaGreen for this system. The results demonstrated that the RSD for the relative fluorescence intensity and migration time was lower than 6.2% and 1.1%, respectively. Smaller apertures improve the RSL but may reduce the theoretical number plate. The system’s limit of detection was 0.05 ng/μL. To validate its practicality, we have successfully separated the PCR products of *P.g* and *Candida albicans* and determined the size of the amplicon by comparing its migration time with that of DNA ladders. Such a system is low cost and easy to assemble and may offer an ideal way for researchers in the third world to develop their own CE system. To make it more versatile, we are developing a high-throughput CE system that can separate samples in 12 capillaries simultaneously.

## Figures and Tables

**Table 1:** Relative standard deviation of peak and migration time.

DNA Size (bp)	50	100	150	200	250	350	450	550
Fluorescence intensity	5.2%	5.1%	4.2%	6.0%	6.1%	5.2%	6.2%	6.0%
Migration time	0.9%	1.1%	0.5%	0.2%	0.2%	0.3%	0.5%	0.6%

## Data Availability

Data will be made available on request.

## Supplementary Materials

The following supporting information can be downloaded at https://www.mdpi.com/article/10.3390/bioengineering12030303/s1, Figure S1: The transmission of the first optical filter; Figure S2: The transmission of the second optical filter.
