# Supplementary material for: Development of a Low-Cost and Easy-Assembly Capillary Electrophoresis System for Separation of DNA
Source: Bioengineering (Basel). 2025 Mar 17;12(3):303. doi: 10.3390/bioengineering12030303 (PMC11939447; doi:10.3390/bioengineering12030303)
Supplement: Supplementary file 1 [file bioengineering-12-00303-s001.zip › bioengineering-3496165-supplementary.pdf]

Development of a Low-Cost and Easy-Assembly Capillary Electrophoresis System for Separation of DNA

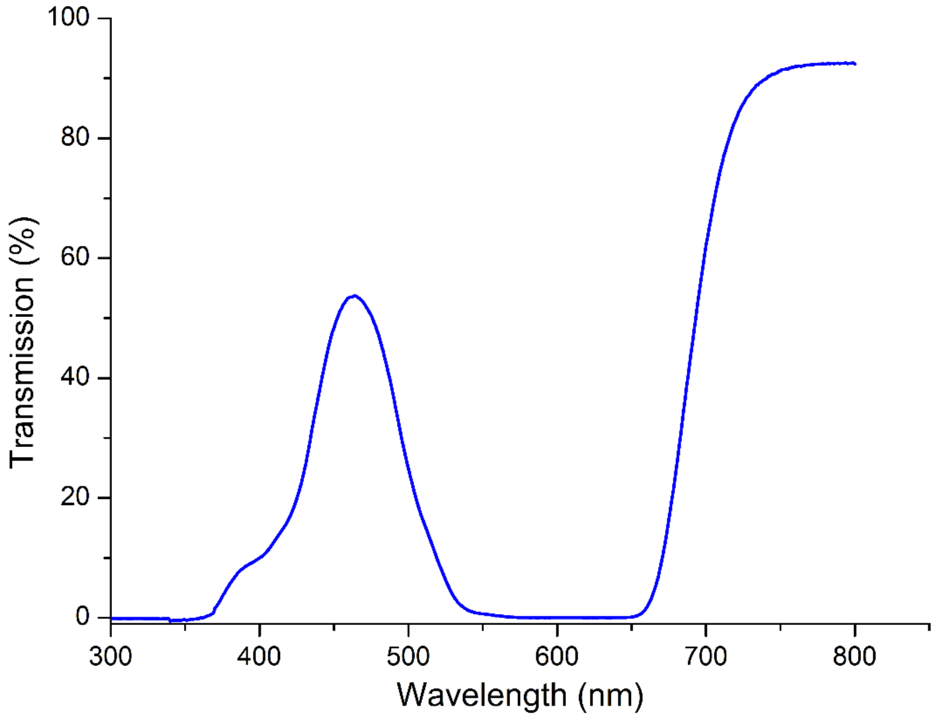

Figure S1. The transmission of the first optical filter.

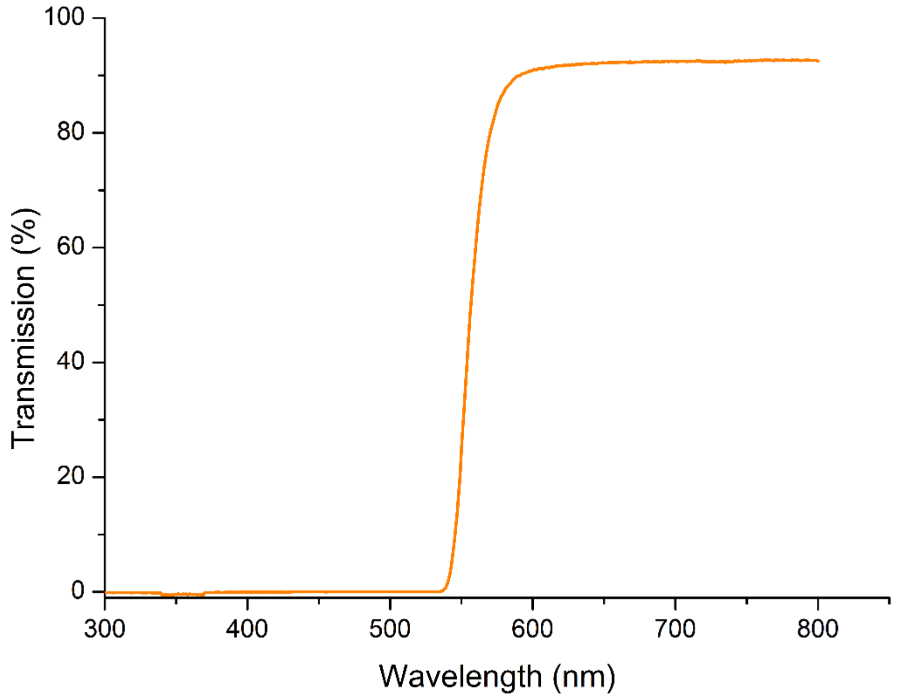

Figure S2. The transmission of the second optical filter.
